# Gastric volvulus in children - a diagnostic problem: two case reports

**DOI:** 10.1186/s13256-016-0934-3

**Published:** 2016-05-31

**Authors:** Ilaria Trecroci, Giuliana Morabito, Claudio Romano, Ignazio Salamone

**Affiliations:** 1 Radiology Department, University of Messina, Policlinico G. Martino, Gazzi, Messina, 98100 Italy; 2 Pediatric Department, University of Messina, Via Consolare Valeria 1, 98125 Messina, Italy

**Keywords:** Gastric volvulus, Vomiting, Radiography, Computed tomography, Pediatrics

## Abstract

**Background:**

Gastric volvulus is a clinically significant cause of acute or recurrent abdominal pain and chronic vomiting in children. Since related clinical symptoms are nonspecific, clinicians often refer to radiologists for a diagnostic evaluation. Early diagnosis is crucial to prevent life-threatening complications of prolonged volvulus, such as intestinal ischemia, infarction, strangulation, necrosis, and perforation that may require immediate surgical treatment. In this report, we describe clinical and radiological criteria for diagnosis of gastric volvulus in children.

**Case presentation:**

We describe two pediatric clinical cases. A 16-month-old female Caucasian child was admitted to our hospital for recurrent postprandial vomiting episodes, which started at 11 months old, associated with failure to thrive. A 9-month-old term-born baby boy was admitted for chronic, recurrent, postprandial vomiting, which started at 7 months of age, with progressive failure to thrive. A barium study allowed definitive diagnosis of chronic organoaxial gastric volvulus.

**Conclusions:**

Gastric volvulus is an extremely rare disorder in the pediatric population. It can be considered a complex clinical condition with regard to the etiology and the management. A nonoperative approach is advisable in the absence of warning signs.

## Background

Gastric volvulus (GV) consists of a pathological rotation of the stomach, greater than 180°, around its axis, causing obstruction of the gastrointestinal tract. It should be differentiated from “gastric torsion”, a simple stomach rotation of less than 180° [1]. Currently, GV can be classified according to many features, helping to recognize it promptly and carry out correct management. It can be classified into acute or chronic form, and into intra-abdominal and thoracic. The latter is more frequent in the neonatal age group and due to the presence of a Bochdalek hernia, or other congenital diaphragmatic defects with dislocation of the stomach in the chest that causes acute respiratory distress and requires early life-saving surgical treatment [2]. It is also possible to distinguish GV into organoaxial, mesenteroaxial and combined types. Organoaxial (OA) volvulus occurs when the stomach rotates along its longitudinal axis, with the greater curvature positioned above and right of the lesser curve. It involves two sites of obstruction, the gastric cardia and the pylorus, and thus represents the type of torsion most apt to strangulate. Mesenteroaxial (MA) volvulus occurs when the stomach rotates along its short axis, with consequent displacement of the antrum above the gastroesophageal junction. Obstruction usually occurs at the mobile pylorus antrum with complete occlusion or strangulation and spontaneous detorsion [3]. Stomach rotation about both its longitudinal and transverse axes is defined as “combined” or “mixed-type (MT)” [4]. OA is much more common than MA and represents about two thirds of GV cases. Based on etiology, GV can be divided into primary (or idiopathic) and secondary. The first being associated with congenital defects, as absence or laxity, of the supporting structures of the stomach (gastrocolic, gastrohepatic, gastrosplenic, gastrophrenic ligaments), or with abnormal gastric distention; the second is associated with abnormalities of other organs (diaphragmatic hernia, wandering spleen, congenital bands) [5]. According to the clinical course, GV can be further subdivided into: acute, often secondary to major defects, and chronic (recurrent or intermittent). Anatomical defects are usually associated with acute GV; on the contrary, the absence or laxity of ligaments that link the stomach are predisposing factors for chronic GV.

Diagnosis of chronic GV may be delayed but should be suspected in children with a clinical history of chronic vomiting, abdominal distension and recurrent pulmonary infection. Borchardt *et al*. have described three main features, known as the Borchardt’s triad, of acute GV, which include unproductive retching, epigastric pain and a passage blockage of nasogastric tube. Productive retching is reported in 71–75 % of the patients [6]. Diagnostic criteria are not always straightforward and there is the need for close collaboration between radiologist and clinicians. In this report, we describe the clinical and radiological criteria for diagnosis of GV in children.

## Case presentation

### Case 1

**Fig. 1:**
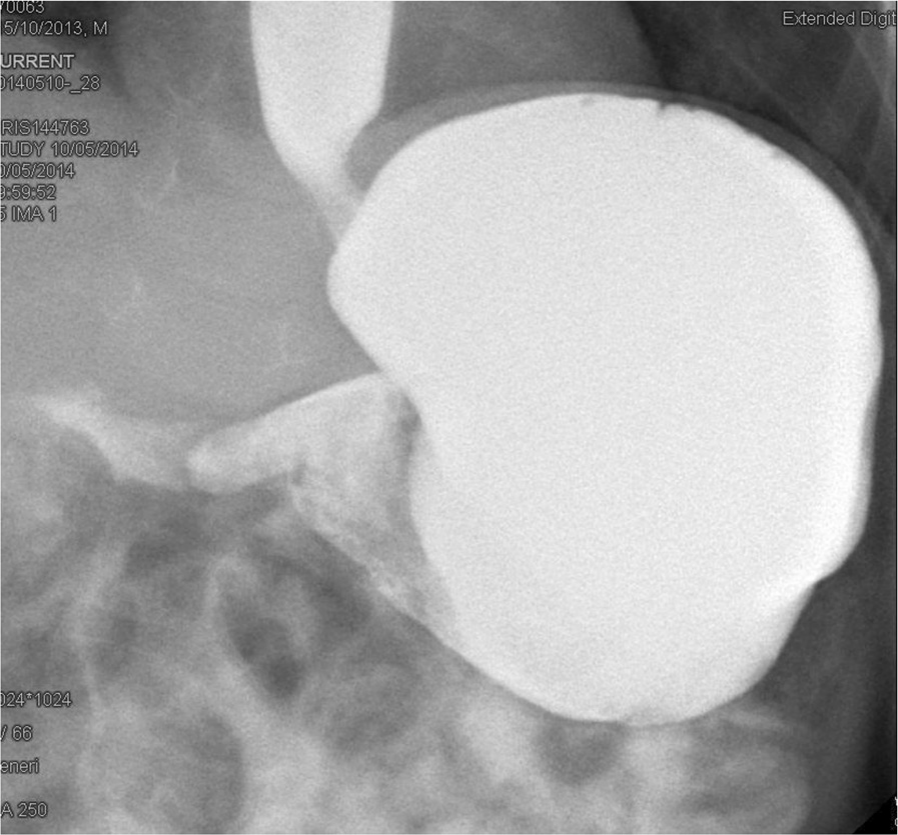
Regular esophageal lumen with opacification of the gastric lumen until the pylorus

A 16-month-old female Caucasian child was admitted to our hospital for recurrent and postprandial vomiting episodes, which started at 11 months of age. A history of gastroesophageal reflux, unresponsive to drug therapy, was present until she was 1 year old, in association with recurrent respiratory infections. On physical examination, her weight was 9.97 kg (less than the fiftieth percentile) and height 82.5 cm (less than the ninety-fifth percentile). Her general condition at admission was good, with abdominal bloating in the absence of organomegaly. A routine basic metabolic panel was performed, along with general functional tests, complete blood count, and C-reactive protein test. All results were normal, in association with plasma amino acids, celiac serology, urinalysis, cytomegalovirus serology, stool test, and parasitological examination of stools. A neurological examination with electroencephalogram and fundus oculi was normal. A barium study showed stomach rotation along a horizontal plane of the stomach, with partial rotation of the greater curvature toward the diaphragmatic side; the stomach was markedly dilated with delayed gastric emptying (Fig. 1). Esophagogastroduodenoscopy was negative for mucosal alterations. A diagnosis of chronic organoaxial GV was advanced with evidence of increasing difficulty in feeding. Due to the persistence of episodes of vomiting, in combination with progressive failure to thrive, gastropexy and Nissen fundoplication were performed. At the 6-month follow-up, the child demonstrated catch-up growth with regular calorie intake.

### Case 2

**Fig. 2:**
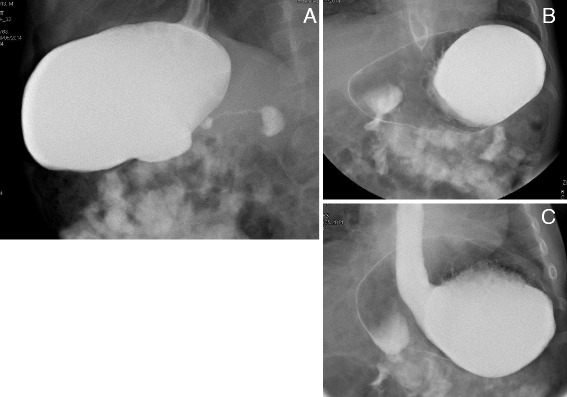
The radiographic study of the transit gastroesophageal barium meal with a projection. Lateral (**a**) and anteroposterior (**b** and **c**) views show marked reduction in the caliber of the pyloric region (**a**), the arrangement of the greater and lesser curvature (**b**) and reflux of contrast up to the middle third of the esophagus

A 9-month-old male Caucasian baby was admitted to our hospital for chronic, recurrent, postprandial vomiting, which started at 7 months of age. He was a term-born baby, at birth his weight was 2.070 kg and during the first days support was required for respiratory distress. Since birth, he was formula fed, underwent gluten-free weaning at 5 months, and was regularly vaccinated. A history of gastroesophageal reflux was reported with regular growth. His symptomatology began at 7 months of age and was characterized by recurrent episodes of postprandial vomiting associated with progressive failure to thrive. On admission, his weight and height were respectively 7.28 kg and 67 cm. A routine basic metabolic panel was performed. Aminoacidemia and aminoaciduria determination was negative. Results of celiac serology tests, thyroid functionality tests, cytomegalovirus serology tests, urinalysis and culture, stool test, and parasitological examination of stools were negative. An electroencephalogram showed normal electrical activity and brain magnetic resonance imaging (MRI) was negative. An ultrasound (US) examination showed gastrectasia and gaseous distension of the large intestine. A radiological study of transit on barium swallow detected an organoaxial volvulus, with the greater curvature lying to the right of the lesser curvature and above it. There was no obstruction to the passage of the contrast agent into the small bowel. On lateral projection, a marked thinning of the pylorus was present (Fig. 2). Also for this patient, after a short attempt of enteral nutrition by nasogastric tube, an anterior gastropexy was performed in association with Nissen fundoplication. The surgical finding reported only one important gastrectasia and a temporary gastrostomy tube was placed. At 30-day follow-up, a significant improvement in his growth was highlighted and after 1 year there was a complete resolution of his symptoms.

## Discussion

GV is defined as a pathological rotation of the stomach. While acute GV is clinically more evident, with severe epigastric pain and vomiting, or respiratory distress, chronic GV can be much more subtle, presenting with nonspecific symptoms such as gastroesophageal reflux, respiratory infection, recurrent abdominal pain, varying from vague to severe, or just with feeding difficulties associated to epigastric distention, with or without nausea and vomiting. Clinical diagnosis of chronic GV is often difficult, due to the variety of clinical presentations, and, especially, submitted to the correct interpretation of radiological findings. Cribbs *et al*. [7] reported a large series of GV in England. The group with acute GV, 69 %, had pathological anomalies (diaphragmatic eventration, intestinal malrotation, and so on). Nonbilious vomiting was the most common presenting symptom of the group with chronic GV. In this casistic, overall 40 % of the patients with chronic GW were treated without surgical intervention. In the past decades, GV has been diagnosed by means of plain radiography [8]. Plain radiography findings, suggestive for GV, included the double bubble sign, abnormal gastric distension despite the presence of a nasogastric tube, a distended stomach lying in a horizontal plane, and a fluid level projecting into the epigastric region [9]. The “double bubble sign”, due to the appearance of two overlapping spherical images in the epigastrium, with elevation of the left hemidiaphragm, clearly displayed on conventional abdomen and chest X-ray, may indicate GV: diagnostic confirmation with a barium meal is usually required. A contrast study (upper gastrointestinal [UGI]) was helpful in confirming the diagnosis of chronic organoaxial gastric volvulus. Another modality of evaluating patients with these clinical signs/symptoms is a computed tomography (CT) scan that can delineate their anatomy much more clearly to avoid a delay in diagnosis that can lead to a life-threatening situation [10]. Both studies have the disadvantage of utilizing ionizing radiation, however obtaining the correct diagnosis in the most effective and efficient manner possible is crucial. Both studies can be used to make the diagnosis, and discussion with the radiologist is encouraged. Together, you can determine the best diagnostic maneuver at your institution and care can be individualized [11]. The mortality rate for acute GV is more than twice in comparison with chronic GV. The possibility of ischemia and perforation is the higher risk in acute gastric volvulus with necrosis, as reported in 5–28 % of cases and the mortality rate is reported in 50 % of cases [7]. Nonsurgical solutions for chronic GV could be considered, including antisecretory therapy, diet modifications (thickening of meals), and posture changes (ensuring patients are upright for feeds and consideration for positional changes with right side down or prone after feeding) [12]. No data are present about the outcome and follow-up of this condition.

## Conclusions

In conclusion, GV is an extremely rare disorder in the pediatric population. It can be considered a complex clinical condition with regard to the etiology and the management. Symptoms are related to the type, extent, and degree of rotation and obstruction, or associated congenital defect. The organoaxial type can be considered most frequent, and clinical symptoms can be acute or chronic. It may be suspected from plain radiography but a UGI series is suggested for the definitive diagnosis. A CT scan of the abdomen is often necessary and most prudent in acute volvulus and it can demonstrate associated congenital gastrointestinal defects (for example, paraesophageal hernia). Management of chronic GV should be carefully individualized, case by case. Anterior gastropexy can be satisfactorily employed, by a gastroscopic and laparoscopic approach. The insertion of a gastrostomy tube can be considered in children with failure to thrive. The resolution of symptoms can be considered the most important outcome after the surgery. The nonoperative approach can be successful in the absence of warning signs such as weight loss, abdominal pain, or worsening of symptoms. The mortality rate for acute gastric volvulus is more than twice that of chronic gastric volvulus. Although there is no data in the literature regarding the follow-up of these patients, the reduction or disappearance of symptoms and good nutritional status can be considered the most important prognostic factors.
